# RPA and Pif1 cooperate to remove G-rich structures at both leading and lagging strand

**DOI:** 10.15698/cst2020.03.214

**Published:** 2020-01-17

**Authors:** Laetitia Maestroni, Julien Audry, Pierre Luciano, Stéphane Coulon, Vincent Géli, Yves Corda

**Affiliations:** 1 Aix-Marseille Univ, Inserm, CNRS, Institut Paoli-Calmettes, CRCM, Marseille, France. Equipe Labellisée par la Ligue Nationale contre le Cancer.

**Keywords:** RPA, Pif1, human minisatellite CEB1, G-rich structures, G-quadruplex

## Abstract

In *Saccharomyces cerevisiae*, the absence of Pif1 helicase induces the instability of G4-containing CEB1 minisatellite during leading strand but not lagging strand replication. We report that RPA and Pif1 cooperate to maintain CEB1 stability when the G4 forming strand is either on the leading or lagging strand templates. At the leading strand, RPA acts in the same pathway as Pif1 to maintain CEB1 stability. Consistent with this result, RPA co-precipitates with Pif1. This association between Pif1 and RPA is affected by the *rfa1-D228Y* mutation that lowers the affinity of RPA in particular for G-rich single-stranded DNA. At the lagging strand, in contrast to *pif1*Δ, the *rfa1-D228Y* mutation strongly increases the frequency of CEB1 rearrangements. We explain that Pif1 is dispensable at the lagging strand DNA by the ability of RPA by itself to prevent formation of stable G-rich secondary structures during lagging strand synthesis. Remarkably, overexpression of Pif1 rescues the instability of CEB1 at the lagging strand in the *rfa1-D228Y* mutant indicating that Pif1 can also act at the lagging strand. We show that the effects of the *rfa1-D228Y* (*rpa1-D223Y* in fission yeast) are conserved in *Schizosaccharomyces pombe*. Finally, we report that RNase H1 interacts in a DNA-dependent manner with RPA in budding yeast, however overexpression of RNase H1 does not rescue CEB1 instability observed in *pif1*Δ and *rfa1-D228Y* mutants. Collectively these results add new insights about the general role of RPA in preventing formation of DNA secondary structures and in coordinating the action of factors aimed at resolving them.

## INTRODUCTION

Replication protein A (RPA) is the major eukaryotic single-stranded DNA (ssDNA) binding protein that consists of 70, 32, and 14 kDa subunits [1]. RPA plays a key role in coordinating DNA synthesis, repair, and DNA damage signalling through binding to single-stranded DNA (ssDNA) intermediates generated during these processes [2]. RPA primarily maintains ssDNA in an unfolded state through different binding modes that are characterized by the length of the interacting ssDNA [3]. Thus, RPA binds to ssDNA with high affinity preventing the formation of DNA secondary structures and annealing of homologous sequences [4]. Among its activities, RPA is involved in the maturation of Okazaki fragments by governing the sequential action of Dna2 and Fen1 [5]. During Pol δ synthesis most flaps generated on the lagging strand, by strand displacement, are normally cleaved by Fen1. However, a minor fraction escapes cleavage [6], and the 5′ ssDNA flaps on Okazaki fragments get extended by Pif1, a 5′ to 3′ helicase, to create substrates for RPA binding that inhibits Fen1′s cleavage [5,7–11]. RPA is next displaced by Dna2 which cleaves the long flap, generating a short flap structure that undergoes cleavage via Fen1 [12–14].

Interestingly, it has been reported *in vitro,* that RPA binds to and unwinds G4 structures in a 5′ to 3′ direction [15]. G4 are polymorphic and consist of four-stranded structures formed at specific G-rich motifs within DNA, RNA, and into R loops, a RNA-DNA hybrid structure, that can eventually lead to genome instability [16–19]. The core of these structures is formed by a square arrangement of four guanines held together by Hoogsteen hydrogen bonds [20, 21]. Under specific conditions, G4 structures are recognized by specific factors and their formation is controlled [22, 23]. However, it has been shown that highly stable G4 structures impede fork progression. Hence, their unwinding by helicases is critical [17, 24, 25]. Many helicases are able to unwind G4 structures *in vitro* such as the RecQ helicases BLM, WRN and Sgs1 and other helicases such as Pif1, FANCJ or RTEL1 [24,26–30]. G4 structures are also targeted by additional proteins that protect them [23] or support the function of an helicase at G4 [22]. In budding yeast, unwinding of G4 is mainly performed by the Pif1 helicase [26]. Indeed, a particular example is the 1.8 kb G4-forming human minisatellite CEB1, a reporter of G4 formation and processing [31, 32]. In cells lacking Pif1, CEB1 is unstable when inserted into *Saccharomyces cerevisiae* genome, near an early origin of replication (ARS305). Instability of CEB1, which consists in 42 motifs of 39 nucleotides arranged as direct repeats, was correlated to the ability of the CEB1 motif to form G4 [31, 33, 34]. Surprisingly, in *pif1*Δ cells CEB1 was unstable only when the G-quadruplex-forming strand was the leading strand template [33]. This result is apparently counterintuitive since ssDNA is mostly formed during the discontinuous synthesis of the lagging strand DNA [35] and because Pif1 has been reported to act at the lagging strand [36] and binds G4 structures located in the lagging strand [22, 37].

We have previously shown in fission yeast that the *rpa1-D223Y* mutation (*rfa1-D228Y* in budding yeast), that exhibits a reduced affinity for ssDNA, impaired lagging strand telomere replication and provoked accumulation of secondary structures [38]. Consistently, expression of ScPif1 rescued the phenotypes associated with the *rpa1-D223Y* mutation [38]. These results suggested that *rpa1-D223Y* cells accumulated G-rich structures at lagging strand telomeres that were resolved by the heterologous expression of ScPif1. In *S. cerevisiae*, RPA subunits are encoded by *RFA1, RFA2*, and *RFA3* genes. The *rfa1-D228Y* mutant (*rpa1-D223Y* in fission yeast) also possesses a lower affinity for ssDNA and reduced ability in removing secondary structure from ssDNA [39, 40]. In this study, we investigated the role of RPA in maintaining the stability of CEB1 when the G-quadruplex-forming strand is either on the leading or lagging strand template. Our results indicate that both RPA and Pif1 cooperate at the leading strand to maintain the stability of CEB1. Consistent with this hypothesis, RPA co-precipitates with Pif1. In contrast to Pif1, RPA is also required to stabilize CEB1 when the G-quadruplex-forming strand is the lagging strand. However, under a situation that compromises RPA binding to ssDNA, overexpression of Pif1 rescued lagging-CEB1 instability, suggesting that Pif1 can unwind G4 at the lagging strand. Interestingly, Mms1 which binds to G-rich/G4 regions and supports the binding of Pif1, is not required to maintain the stability of CEB1. Based on these data we propose a model in which RPA facilitates Pif1 action at the leading strand DNA to unwind G4 while enriched RPA at the lagging strand DNA prevents by itself formation of stable G4, explaining why Pif1 is dispensable. We extended the role of RPA in preventing non-templated DNA single-stranded structure by showing that RPA interacts with RNAse H1 in a DNA-dependent manner in *S. cerevisiae*, as previously reported in human cells [41]. However, we found that overexpressing RNAse H1 did not restore CEB1 stability in both *pif1*Δ and *rfa1-D228Y* mutants, suggesting that CEB1 instability is not due to R-Loop formation arising during transcription.

## RESULTS

### The *rfa1-D228Y* mutation affects both the leading-CEB1 and lagging-CEB1

**Figure 1:**
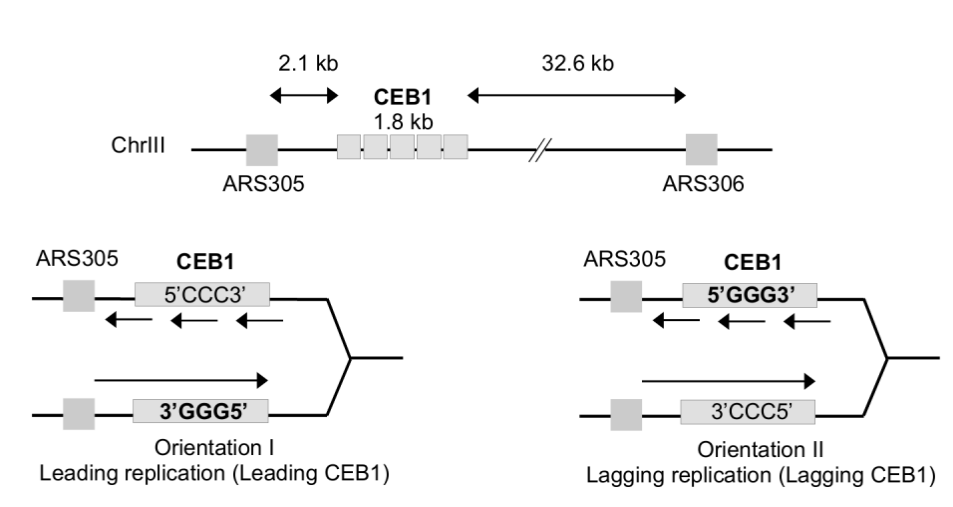
FIGURE 1: Map of the CEB1 insertion within chromosome III at 2.1 kb from the ARS305. In orientation I, the G-quadruplex-forming strand is the template of the leading polymerase (leading CEB1). In orientation II, the G-quadruplex-forming strand is the template of the lagging polymerase (lagging CEB1).

As mentioned above, the G-rich minisatellite CEB1 can be considered as a reporter of G4 formation and processing [31, 32]. We used strains previously constructed in A. Nicolas' laboratory in which the 1.8 kb CEB1 is inserted in both directions at 2.1 kb of *ARS305* and 32.6 kb away from ARS306 allowing to primarily replicate CEB1 only from the proximal ARS305 origin (**Figure 1**). Depending on the orientation of CEB1 insertion, the G4-forming strand will be replicated by the leading or the lagging machinery [33]. We will name leading-CEB1 or lagging-CEB1 to indicate the machinery that replicates the G4-forming strand. Previous results demonstrated that the helicase activity of Pif1 was required to stabilize the leading-CEB1 but that Pif1 was dispensable for the stability of the lagging-CEB1 suggesting the existence of different mechanisms to resolve G4 when the G4-forming strand is replicated by the leading or the lagging machinery [33].

**Figure 2:**
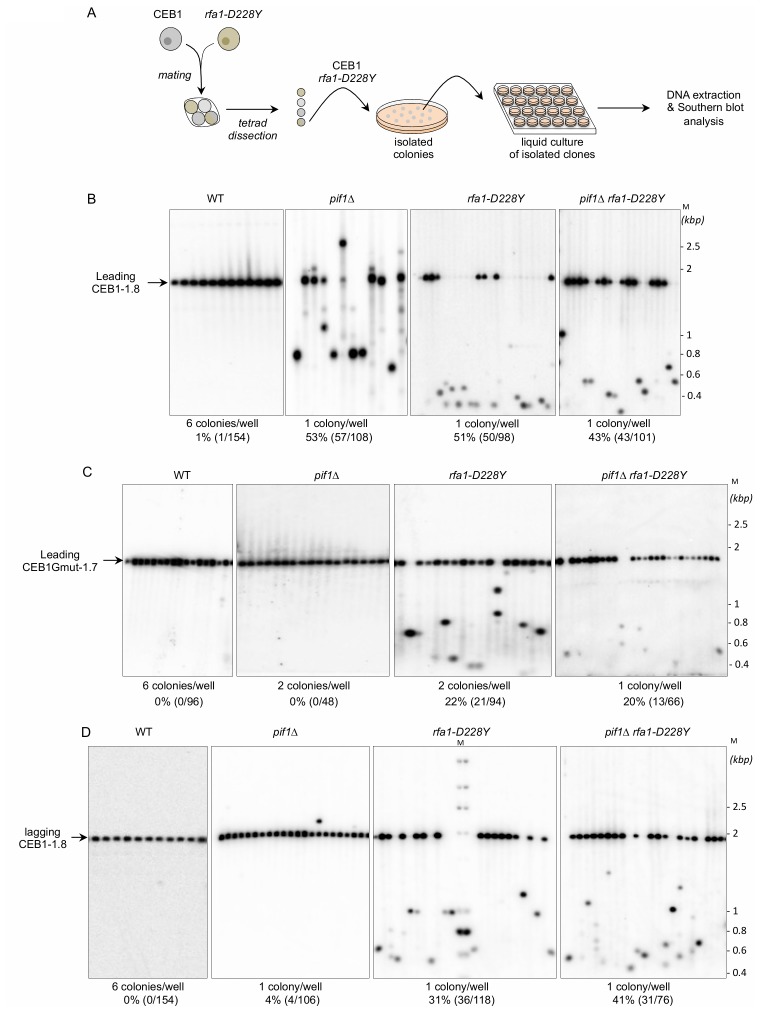
FIGURE 2: RPA is required to stabilize CEB1. **(A)** Experimental scheme. Yeast strains of interest are mated and the resulting diploid sporulated and dissected. After identification of spore-colonies of interest, the spore-colony is plated on media to obtain isolated colonies. Individual colonies are placed in liquid culture until stationary growth phase. Genomic DNAs are extracted and analysed by Southern blot. **(B)** RPA is required to stabilize CEB1 when the G4-forming strand is replicated by the leading polymerase. Genomic DNAs from yeast cells bearing the leading-CEB1 were digested by *Apa*I and *Xho*I, and southern blotted. Membranes were hybridized with the CEB1-0.6 probe. **(C)** Genomic DNAs from yeast cells containing the leading-CEB1Gmut-1.7 were digested by *Apa*I and *Sac*II, and southern blotted. The membranes were hybridized with the CEB1Gmut-1.7 probe. **(D)** In contrast to Pif1, RPA is required to stabilize CEB1 when the G4-forming strand is replicated by the lagging polymerase. Genomic DNAs from yeast cells bearing the lagging-CEB1 were digested by *Apa*I and *Nco*I, and southern blotted. Membranes were hybridized with the CEB1-0.6 probe. M: ladder DNA serving as size standard (kbp). The number of colonies analysed per well, the percentage of rearrangement frequencies, and the total numbers of colonies are indicated in Table 1.

We thought to test the role of RPA in the CEB1 stability by determining the effect of the *rfa1-D228Y* mutant whose DNA-binding activity, in particular to G-rich ssDNA, is compromised [38, 39]. We used an experimental scheme (**Figure 2A**) adapted from Lopes *et al.* [33]. Briefly, tetrads obtained from the sporulation of heterozygous diploids were dissected. Spore colonies carrying the appropriated mutation were then resuspended in water and streaked on YPD plates in order to obtain about 200 isolated colonies. Colonies were inoculated in YPD liquid cultures, grown to saturation, and genomic DNAs were prepared from the liquid cultures. The size of CEB1 was monitored by Southern blot. We calculated the frequency of instability by monitoring CEB1 size variations (contractions and expansions). We considered that CEB1 was unstable when the intensity of the band(s) was superior to the one of the parental band. We therefore partially discriminated between early or late events of CEB1 rearrangements.

We first analysed the stability of the leading-CEB1 (orientation I) in wild type (WT), *pif1*Δ, *rfa1-D228Y*, and *pif1*Δ *rfa1-D228Y* cells (**Figure 2B**). As shown in representative gels and as previously reported [31], the leading-CEB1 was stable in the WT strain but extremely unstable in the *pif1*Δ mutant (53% of rearrangements) (**Figure 2B, Table 1**). In most cases, we obtained contractions except in one case (**Figure 2B**, second panel). In the *rfa-1D228Y* mutant, leading-CEB1 was also unstable (51% of rearranged colonies). Interestingly, the double mutant *pif1*Δ *rfa1-D228Y* exhibited a level of instability in a similar range of the two single mutants (43%), suggesting that RPA and Pif1 could act in similar pathways to unwind G4 and stabilize CEB1 when the G4-forming strand is replicated by the leading polymerase (**Figure 2B**).

**TABLE 1.** Rearrangement frequencies of CEB1 placed near ARS305 in both orientations in WT and mutant strains.

Minisatellite	Genotype	Orientation I	Orientation II
**CEB1-1.8**	WT	1/154 (1%)	0/154 (0%)
	*pif1Δ*	57/108 (53%)	4/106 (4%)
	*rfa1-D228Y*	50/98 (51%)	36/118 (31%)
	*pif1Δ rfa1-D228Y*	43/101 (43%)	31/76 (41%)
	WT *GAL::PIF1*	0/44 (0%)	0/43 (0%)
	*rfa1-D228Y GAL::PIF1*	12/39 (31%)	2/43 (5%)
	*mms1Δ*	0/116 (0%)	3/116 (3%)
	*rtt105Δ*	116/116 (100%)	116/116 (100%)
	WT *GAL::RNH1*	1/58 (2%)	0/58 (0%)
	*pif1Δ GAL::RNH1*	30/58 (52%)	ND
	*rtt105Δ GAL::RNH1*	58/58 (100%)	58/58 (100%)
	*rfa1-D228Y GAL::RNH1*	39/87 (45%)	29/87 (33%)
**CEB1-Gmut-1.7**	WT	0/96 (0%)	ND
	*pif1Δ*	0/48 (0%)	ND
	*rfa1-D228Y*	21/94 (22%)	ND
	*pif1Δ rfa1-D228Y*	13/66 (20%)	ND

ND – not determined.

We next analysed the effect of *pif1*Δ, *rfa1-D228Y*, and *pif1*Δ *rfa1-D228Y* on the stability of CEB1-Gmut, a version of CEB1 mutated for its G-quadruplex-forming sequences [31, 32]. The CEB1-Gmut was inserted at the same location and in the same orientation as the leading-CEB1. As reported, the CEB1-Gmut was stable in *pif1*Δ cells [33]. In the *rfa1-D228Y* and *pif1*Δ *rfa1-D228Y* mutants, the % of CEB1-Gmut rearrangements dropped to 22% and 20%, respectively (**Figure 2C**). We concluded that the high leading-CEB1 instability observed in the *rfa1-D228Y* mutant partly relies on its ability to form G4. However, the residual CEB1-Gmut instability suggests that the *rfa1-D228Y* mutation affects the replication of the 42 repeated motifs of CEB1-mut irrespectively of the presence of G4.

We next examined the stability of the lagging-CEB1 (orientation II) in the same mutants as above. As reported [33], deleting *PIF1* had no effect on the stability of the lagging-CEB1 (**Figure 2D**). In contrast, the frequency of rearrangements of the lagging-CEB1 was clearly increased in the *rfa1-D228Y* mutant (31%). These results suggest that when G4-forming sequences are localized at the lagging strand, RPA prevents the instability of CEB1. They are consistent with the known enrichment of RPA at the lagging strand [35]. Interestingly, despite the fact that *pif1*Δ did not affect the stability of the lagging-CEB1, combining *pif1*Δ with *rfa1-D228Y*, slightly aggravated the CEB1 instability phenotype of the single *rfa1-D228Y* mutant (41%). This result suggests that Pif1 can be active at the lagging-CEB1.

### Overexpression of Pif1 in *rfa1-D228Y* cells rescues lagging-CEB1 instability

**Figure 3:**
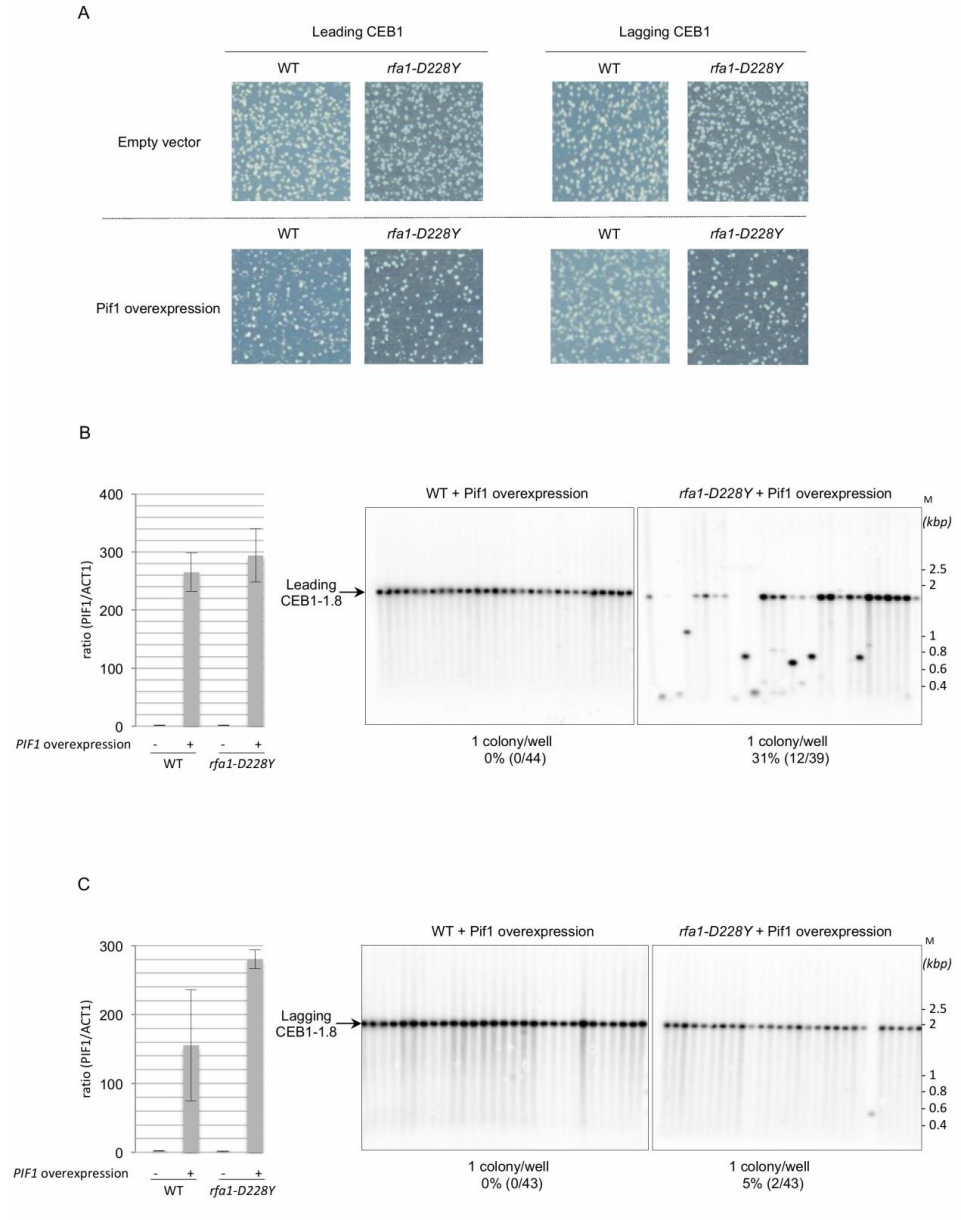
FIGURE 3: Pif1 overexpression rescues lagging-CEB1 instability in the *rfa1-D228Y* mutant. **(A)** Colonies containing the leading- or lagging-CEB1, and overexpressing Pif1 (Pif1 overexpression) or not (Empty vector) were plated on galactose medium and subsequently incubated at 30°C. **(B)** Left: cDNA was prepared from the indicated strains. The ration of *PIF1* transcript to that of *ACT1* was determined using qPCR. (-): Empty vector, (+): Pif1 overexpression. Right, genomic DNAs from wild-type (WT) or *rfa1-D228Y* cells overexpressing Pif1 (Pif1 overexpression) and containing the leading-CEB1 were treated as in Figure 2. M: ladder DNA. **(C)** Left: cDNA was prepared from the indicated strains. The ration of *PIF1* transcript to that of *ACT1* was determined using qPCR. (-): Empty vector, (+): Pif1 overexpression. Right, genomic DNAs from wild-type (WT) or *rfa1-D228Y* cells overexpressing Pif1 (Pif1 overexpression) and containing the lagging-CEB1 were treated as in Figure 2. M: ladder DNA. Yeast strains were grown in medium containing galactose (2%). The number of colonies analysed per well, the percentage of rearrangement frequencies, and the total numbers of colonies are indicated in Table 1.

To further understand the functional interaction between RPA and Pif1 in CEB1 instability, we overexpressed Pif1 in *rfa1-D228Y* cells bearing leading-CEB1 or lagging-CEB1. Because strong Pif1 overexpression impairs cell viability [42], we used a low-copy centromeric plasmid in which the nuclear form of Pif1 is under the control of the *GAL1* promoter [43]. We first noticed that Pif1 overexpression was slightly deleterious in *rfa1-D228Y* cells (**Figure 3A**). To estimate Pif1 overexpression we measured the expression of *PIF1* by reverse transcription followed by quantitative PCR. Our data indicate that *PIF1* is overexpressed in a similar level in WT and *rfa1-D228Y* cells expressing *PIF1* under the control of the *GAL1* promoter (**Figure 3B**, left and **3C**, left). Instability of both leading- and lagging-CEB1 was tested in *rfa1-D228Y* cells grown in galactose (SGal) allowing the overexpression of Pif1. We found that Pif1 overexpression had only a modest effect on leading-CEB1 stability in *rfa1-D228Y* cells while it had no effect in WT cells (**Figure 3B**, right). Surprisingly, Pif1 overexpression completely rescued the instability of lagging-CEB1 in *rfa1-D228Y* cells (**Figure 3C**, right). We concluded that when the G4-forming strand was replicated by the lagging machinery in cells in which the ssDNA binding activity of RPA is compromised, overexpressed Pif1 could act at the lagging strand to unwind G4.

### The *rfa1-D228Y* mutation affects the interaction between Pif1 and RPA

**Figure 4:**
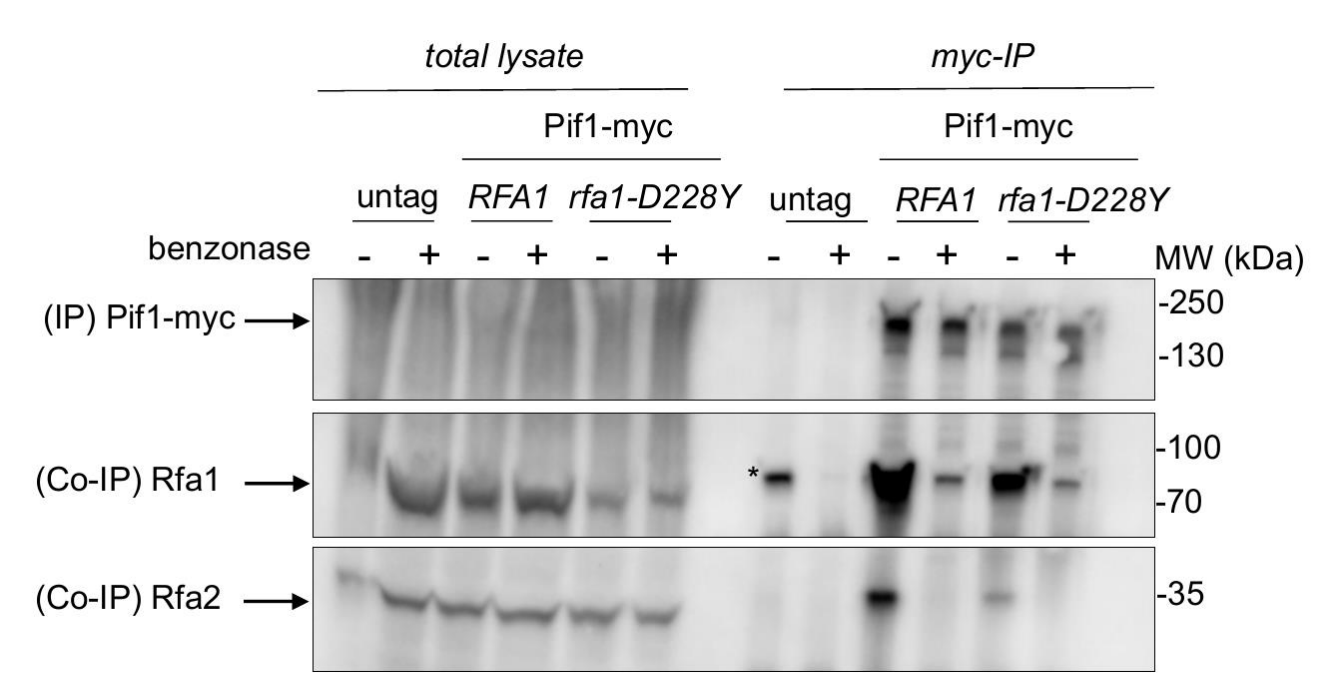
FIGURE 4: The *rfa1-D228Y* mutation affects the interaction between Pif1 and RPA. Co-immunoprecipitation experiments were performed in triplicate. Pif1-myc is immunoprecipitated with an anti-Myc antibody (9E10). The presence of RPA in the Pif1-myc IP is monitored with an anti-Rfa1 or an anti-Rfa2 antibody. (+): cell extracts treated with benzonase. The asterisk (*) indicates a non-specific band. MW: molecular weight.

Our results indicate that RPA and Pif1 could cooperate to stabilize the leading-CEB1 and the lagging-CEB1. This prompted us to test whether Pif1 interacts with RPA. Pif1-myc was immunoprecipitated with anti-Myc antibody and tested for the presence of RPA with a polyclonal antibody directed against Rfa1 or against Rfa2 (**Figure 4**). Pif1-myc was efficiently co-immunoprecipitated with both Rfa1 and Rfa2 but to a lesser extend with *rfa1-D228Y*. To address whether the robust Co-immunoprecipitations (Co-IP) of Pif1-myc with Rfa1 and Rfa2 were dependent on the presence of DNA, DNase1 was added in the lysate. As shown in **Figure 4**, DNA digestion affected the Co-IP suggesting that the presence of DNA is required for the robust co-precipitation of Pif1 and RPA. These results suggest that the robust association of Pif1 with RPA relies on specific structures on the DNA. We think that the interaction between Pif1 and RPA is not only due to unspecific interactions mediated by DNA since the interaction between Pif1 and *rfa1-228Y* is lost despite the fact that the *rfa1-D228Y* still binds to DNA, although with lower affinity [38].

### Mms1 is not required to maintain the leading-CEB1 and the lagging-CEB1 stability

**Figure 5:**
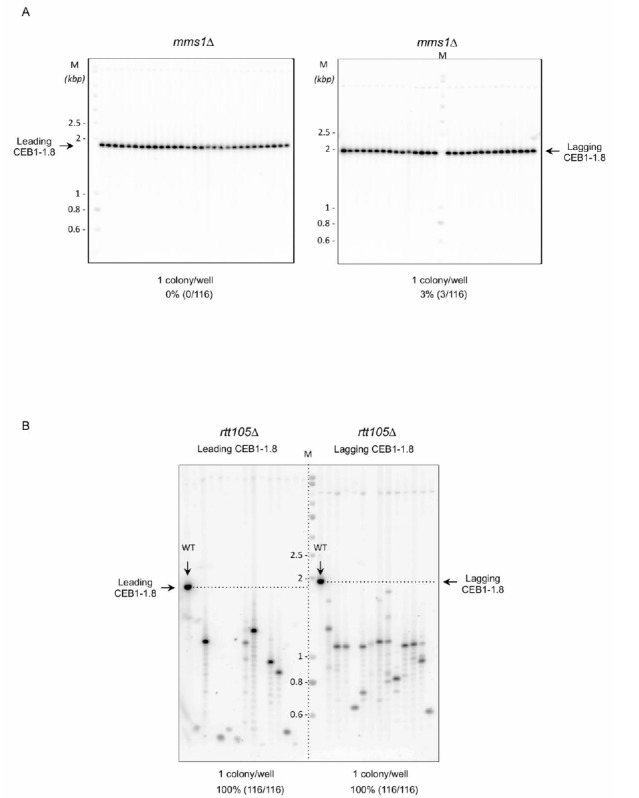
FIGURE 5: Importance of Mms1 and Rtt105 on CEB1 stability. **(A)** Mms1 is not required to stabilize CEB1. Left, genomic DNAs from *mms1*Δ yeast cells bearing the leading-CEB1 were digested by *Apa*I and *Xho*I and southern blotted. Right, genomic DNAs from *mms1*Δ yeast cells bearing the lagging-CEB1 were digested by *Apa*I and *Nco*I and southern blotted. Membranes were hybridized with the CEB1-0.6 probe. **(B)** Rtt105 is required to stabilize both leading-CEB1 and lagging-CEB1. Left, genomic DNAs from *rtt105*Δ yeast cells bearing the leading-CEB1 were digested by *Apa*I and *Xho*I and southern blotted. Right, genomic DNAs from *rtt105*Δ yeast cells bearing the lagging-CEB1 were digested by *Apa*I and *Nco*I and southern blotted. Membranes were hybridized with the CEB1-0.6 probe. WT: wild-type genomic DNA. The arrows show the position of stable leading-CEB1 (left) and stable lagging-CEB1 (right). The horizontal dashed lines indicate the theoretical position of stable CEB1. M: ladder DNA serving as size standard (kbp). The number of colonies analysed per well, the percentage of rearrangement frequencies, and the total numbers of colonies are indicated in Table 1.

Mms1 supports Pif1 helicase binding to G4 structures [22]. We thus analysed the importance of Mms1 in CEB1 stability. We found that in *mms1*Δ cells, leading-CEB1 and lagging-CEB1 were rather stable, showing 0% (0/116) and 3% (3/116) of rearrangements, respectively (**Figure 5A, Table 1**). These results show that Mms1 is not required for the stability of both leading-CEB1 and lagging-CEB1. They suggest that CEB1 minisatellites are not targeted by Mms1. Moreover, they indicate that Mms1 does not support Pif1 function at CEB1 and, together with our previous results showing that Pif1 and RPA interact, suggest that RPA could contribute to the recruitment of Pif1 to CEB1, likely by directly recruiting Pif1.

### Rtt105 is required to stabilize both the leading-CEB1 and lagging-CEB1

Rtt105 functions as an RPA chaperone that escorts RPA to the nucleus and facilitates RPA loading onto ssDNA [44]. Consequently, *RTT105* inactivation reduces the association of RPA with ssDNA generated during DNA transactions and affects multiple RPA functions [44, 45]. We examined the importance of Rtt105 on the stability of the leading-CEB1 and lagging-CEB1. We found that CEB1 is extremely unstable in *rtt105*Δ cells. Remarkably, we found that the frequency of rearrangements reaches 100% in both leading- and lagging-CEB1 (**Figure 5B, Table 1**). These results reveal the importance of *RTT105* in promoting the replication of G4-forming CEB1 minisatellite during leading and lagging strand synthesis and more generally, to maintain genome stability. They confirm our previous results obtained with *rfa1-D228Y* mutant indicating that RPA is crucial to remove G-rich structures at both leading and lagging strand.

### RNase H1 interacts with RPA but its overexpression doesn't rescue CEB1 instability in the absence of Pif1 or reduced levels of RPA

**Figure 6:**
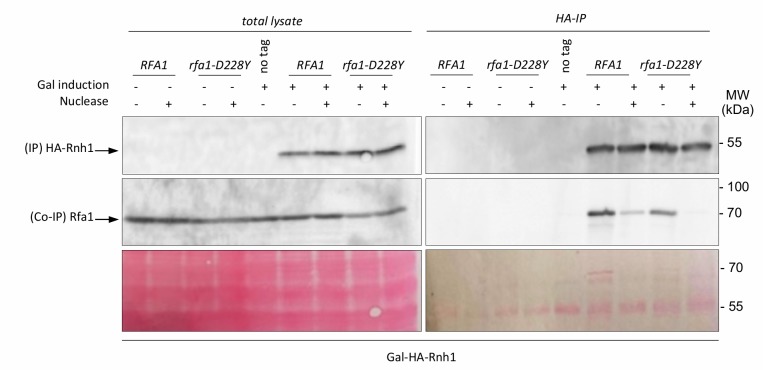
FIGURE 6: Rnh1 interacts with RPA. Co-immunoprecipitation experiments were performed in triplicate. HA-Rnh1 has been overexpressed by galactose addition and immunoprecipitated with an anti-HA antibody. The presence of RPA in the HA-Rnh1 IP is monitored with an anti-Rfa1 antibody. Cell extracts treated with nuclease (Thermofisher) are indicated (+). Yeast strains were grown in medium containing 2% glucose (-) or 2% galactose (+) when mentioned. Total proteins on the membrane were stained with Ponceau S as a loading control (Bottom). MW: molecular weight.

Transcription by RNA polymerase can form a three-stranded structure called R-loop [46], which can facilitate or stabilize secondary structure formation in the exposed ssDNA [16, 47, 48]. Thus, G4 structures may also result from exposure of the G4 forming strand by formation on the other strand of RNA:DNA hybrids. This may create a complex structure involving G4 DNA on one strand and a RNA:DNA hybrid on the other strand [49]. Interestingly, Pif1 has been proposed to have a patrolling role that removes any G4 or RNA/DNA structure [50]. Indeed, Pif1 regulates R-loop formation at specific genomic loci [51] and potentially complements RNAse H for R-loop resolution [52]. On the other side, systematic analysis of protein complexes in *S. cerevisiae* have shown that RPA interacts with RNase H1 [53]. Finally, RPA was recently proposed to act as a sensor of R-loop in human cells and to recruit and stimulate RNase H1 to counteract R-loops [41]. To further document the potential cooperation of RPA with Pif1, we first tested whether RPA interacts with RNase H1 in a DNA-dependent manner in budding yeast. Expression of HA-Rnh1 was induced by the addition of galactose and its interaction with Rfa1 was probed by Co-IP. The results shown in **Figure 6** indicate that Rnh1 interacts with RPA in a way that is stimulated by the presence of DNA, thereby extending the results of Gavin *et al.* [53] and Nguyen *et al.* [41]. Interestingly, we found that the *rfa1-D228Y* mutant had a less efficient ability to bind Rnh1 (**Figure 6**). These results suggest that the leading-CEB1 instability observed on the leading strand in absence of Pif1 and by reducing the association of RPA with ssDNA (*rfa1-D228Y* and *rtt105*Δ mutants) could be the consequence of the presence of R-Loops. To determine if R-loops were responsible of CEB1 instability we analysed the stability of the leading-CEB1 and lagging-CEB1 in WT, *pif1*Δ, *rfa1-D228Y*, and *rtt105*Δ cells overexpressing RNase H1, the enzyme responsible to resolve R-loops. We found that RNase H1 overexpression did not affect CEB1 stability in WT cells, and did not rescue CEB1 stability in *pif1*Δ, *rfa1-D228Y*, and *rtt105*Δ mutants (**Table 1**). Taken together, our data suggest that the instability observed in absence of Pif1 and reduced levels of RPA is not primarily related to R-loop formation.

### The *rpa1-D223Y* mutation affects the stability of CEB25-L1T in fission yeast

**Figure 7:**
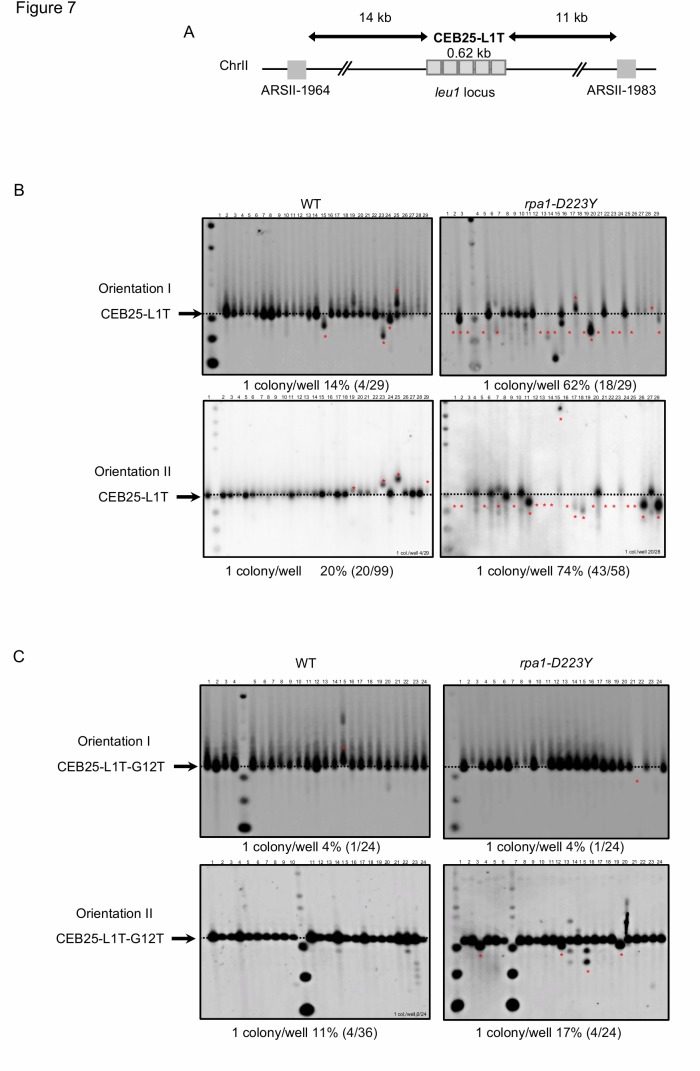
FIGURE 7: RPA is required to stabilize CEB25-L1T in fission yeast. **(A)** Map of the CEB25-L1T insertion within chromosome II at 14 kb from the ARS-II-1964 and at 11 kb from the ARS-II-1983. **(B)** Genomic DNA from cells containing CEB25-L1T in orientation 1 and 2 was digested by *Pvu*II and southern blotted. Membranes were hybridized with CEB25 probe. **(C)** Genomic DNA from cells containing mutated CEB25-L1T-G12T in orientation 1 and 2 was digested by *Pvu*II and southern blotted. Membranes were hybridized with a CEB25-L1T probe. The number of colonies analysed per well, the percentage of rearrangement frequencies, and the total number of colonies are indicated. Red stars mark unstable events. We calculated the frequency of instability by monitoring the size and the intensity of the CEB25. We considered that CEB25 was unstable when the intensity of the short or high band was superior to the one of the parental band or when the band disappears. The number of colonies analysed per well, the percentage of rearrangement frequencies, and the total numbers of colonies are indicated in Table 1.

Because the *rpa1-D223Y* mutation impaired replication of the G-rich lagging strand telomere in *Schizosaccharomyces pombe*, we investigated the stability of CEB in fission yeast. We took advantage of the minisatellite CEB25-L1T which contains 14 repeats of a 44 nucleotides-sequence (0.62 kb) that form a stable G4 [34, 54]. In this L1T version, the loop of the repeated sequence of CEB25 has been reduced to one thymine nucleotide. The shortening of the loop increases its thermal stability in correlation with the *in vivo* instability [54]. As a control the CEB25-L1T-G12T has been used in which the guanine at the 12^th^ position of the repeated sequence has been mutated into thymine, preventing the formation of G4. We introduced the CEB25-L1T and CEB25-L1T-G12T in both orientations into the genome of yeast cells at the *leu1* locus at chromosome 2. The *leu1* locus is located in between ARS-II-1964 and ARS-II-1983 at a distance of 14 kb and 11 kb from CEB25, respectively (**Figure 7A**). These two ARS have a relatively low firing efficiency, 32% and 13%, respectively [55]. We monitored in fission yeast the instability of CEB25 in both orientations although this genomic context does not allow to clearly distinguish whether the G4-forming sequence is replicated by the leading or the lagging replication machinery.

As depicted in **Figure 2A**, the size of CEB25-L1T was monitored by Southern blot and frequency of instability was calculated in both WT and *rpa1-D223Y* strains. The level of instability of CEB25-L1T was 14% and 20% in the WT (**Figure 7B**, left). In the *rpa1-D223Y* mutant, this level increased up to 62% and 74%, respectively (**Figure 7B**, right). Expectedly, the instability of CEB25-L1T-G12T that cannot form G4 was reduced to 4% for leading replication and 17% for lagging replication (**Figure 7C**). These results clearly show that like in budding yeast the RPA complex plays an important role in the replication and stability of G4-forming sequences. Furthermore, the fact that fission yeast lacks a functional homolog of Pif1 helicase, the Pfh1 helicase behaving more closely like the budding yeast Rrm3 helicase [56], may account for the prominent role of RPA complex in G4 unwinding in fission yeast.

## DISCUSSION

In *S. cerevisiae*, Pif1 helicase processes G-rich secondary structures thereby preventing deleterious events that may lead to DNA breaks [33,38,57]. *In vitro*, Pif1 binds tightly to G4 structures and unwinds them very efficiently [26,58].

Previous studies demonstrated that Pif1 prevents the formation of G-quadruplex-dependent CEB1 internal rearrangements during leading strand, but not lagging strand replication [33]. Here, we disclose that RPA cooperates with Pif1 to remove G-quadruplex structures at both leading and lagging strand. We found that the *rfa1-D228Y* mutation increases the frequency of CEB1 rearrangements when the G-quadruplex forming strand is replicated by the leading polymerase. The level of rearrangements is similar to the level observed in *pif1*Δ cells. We report that mutating G-quadruplex-forming sequences strongly decreasesthe instability of CEB1 in *rfa1-D228Y* while it totally abolishes the instability of CEB1 in *pif1*Δ cells. Contrary to *pif1*Δ cells, in *rfa1-D228Y* cells the CEB1 instability does not completely rely on the G-quadruplex-forming sequence of CEB1, indicating that the instability arising in this mutant partially results from G-quadruplex-independent ssDNA-containing secondary structures. We obtained similar results with the double *pif1*Δ *rfa1-D228Y* mutant. Consistent with these results, Pif1 overexpression suppresses the instability of *rfa1-D228Y* mutant in the same proportion as G-mutated CEB1 motif. We therefore propose that RPA cooperates with Pif1 to resolved G-quadruplexes during leading strand replication.

**Figure 8:**
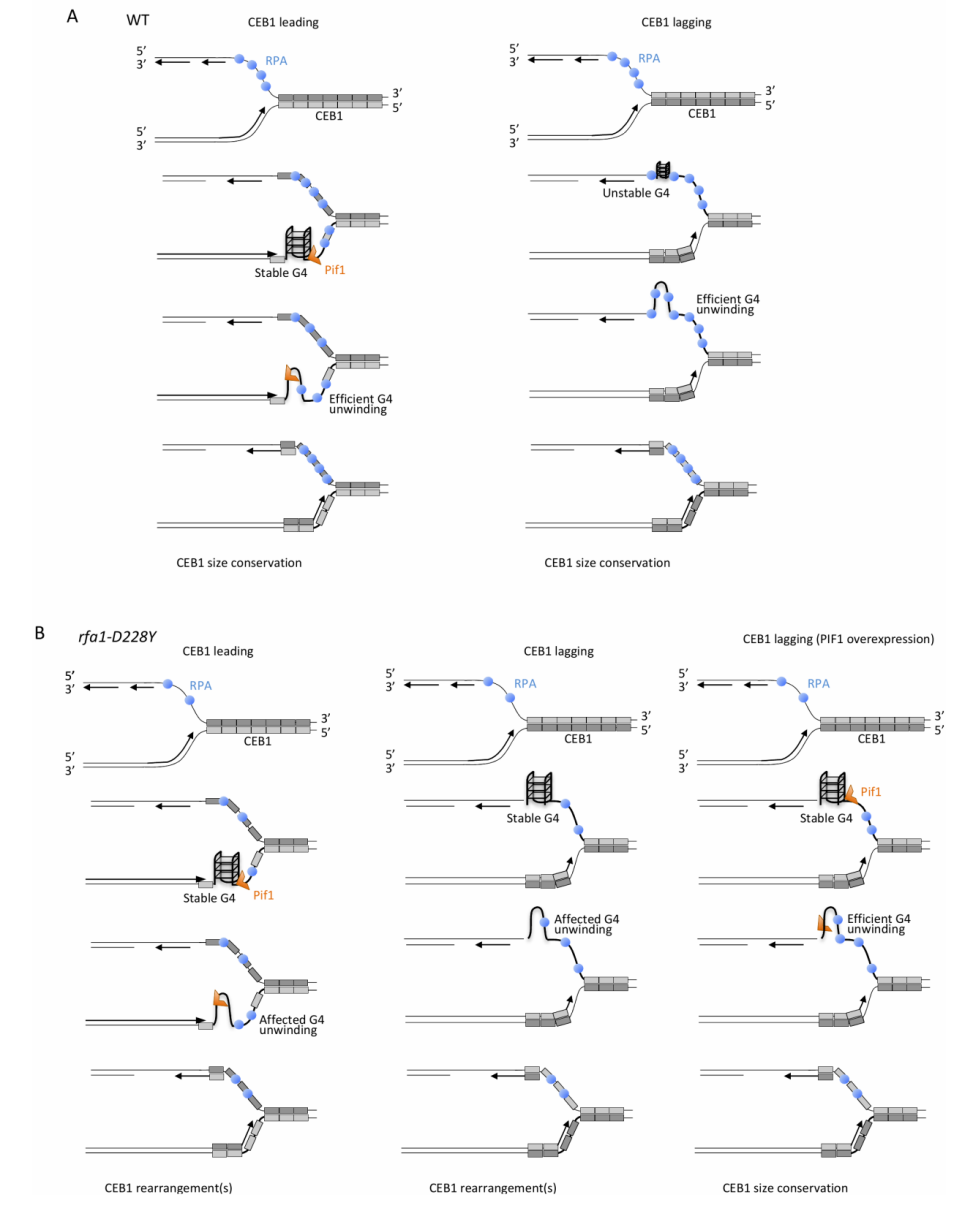
FIGURE 8: Mechanistic model for the unfolding of CEB1-G-rich structures by RPA and Pif1. **(A)** In WT cells, when the G-rich strand (light grey) is on leading strand (left part, CEB1 leading), stable G-quadruplexes structures are efficiently removed by Pif1, as proposed by Lopes and colleagues [33]. At the leading strand, RPA may cooperate with Pif1 either by preventing the refolding of the G4 structure or by recruiting Pif1 to the leading strand. When the G-rich strand (light grey) is replicated by the lagging polymerase (right part, CEB1 lagging) binding of RPA to the G-rich strand prevents formation of stable G-rich secondary structures. In this context, Pif1 is dispensable. **(B)** In the *rfa1-D228Y* mutant, at the leading strand (left part), the reduced affinity of RPA for G-rich ssDNA either reduce the ability of RPA to prevent the refolding of the G4 structure or decrease Pif1 recruitment. The decrease in RPA affects G4-unwinding and leads to the formation of CEB1 rearrangements. At the lagging strand (middle part), the decrease affinity of RPA for G-rich ssDNA facilitates the formation of stable G-rich structures, affects their unwinding, and generates CEB1 instability. In such a case, overexpression of Pif1 (right part) can efficiently unwind G-rich structures, leading to CEB1 size conservation.

In contrast to Pif1, which is not required for CEB1 stability during lagging strand replication, we found that RPA has a prominence in maintaining lagging CEB1 stability. This finding points out the differential behaviour of the *pif1*Δ and *rfa1-D228Y* mutations according to the direction of replication. However, our results indicate that Pif1 overexpression drastically reduced the instability observed at the lagging strand in the *rfa1-D228Y* mutant whose ssDNA binding activity of RPA is compromised, in particular to G-rich regions [38]. Finally, we report that Mms1, which binds G4-structures and aids Pif1 binding to these structures is not required for CEB1 stability at both leading and lagging strands. In contrast Rtt105 that promotes RPA nuclear import, and RPA-ssDNA complex formation at replication forks is required to stabilize CEB1 inserted in both orientations. Collectively these results indicate that Pif1 and RPA cooperate to remove G-quadruplex structures at both leading and lagging strand (**Figure 8**).

Several interpretations can be invoked to explain the difference in CEB1 stability observed in *pif1*Δ cells during leading and lagging replication. It is possible that RPA recruits Pif1. We think that the difference in Pif1 requirement at leading and lagging strands is related to RPA function. At the leading strand, both Pif1 helicase and RPA are required. However, our results showing that mutated CEB1 (which are no longer able to form G4-strutures) and Pif1 overexpression both only partially rescue instability in *rfa1-D228Y* cells reveal higher requirement of Pif1 compared to RPA to maintain CEB1 stability at the leading strand. Interestingly the roles are inverted between RPA and Pif1 at the lagging strand. Because contrary to the leading strand the lagging strand contains longer stretch of ssDNA between elongating Okazaki fragments, RPA is present at a higher concentration at the lagging strand [59]. Consequently, by preventing formation/folding of G-quadruplex structures at the exposed ssDNA, RPA is likely to directly contribute to CEB1 stability independently of Pif1 at the lagging strand. Because *rfa1-D228Y* possesses a lower affinity for ssDNA and a reduced ability in preventing and removing secondary structures from ssDNA, in *rfa1-D228Y* cells CEB1 becomes unstable. In that situation Pif1, which is not initially required to remove G-quadruplex at the lagging strand, is now required. Our results showing that the Pif1 overexpression almost fully rescues the stability of CEB1 in *rfa1-D228Y* mutant at the lagging strand suggest that CEB1-G- quadruplexes are formed in this mutant and can be processed by Pif1 indicating that RPA and Pif1 have redundant G-quadruplex-processing activities at the lagging strand and cooperate (**Figure 8**).

Pif1 binds to G4 motifs with no preference for leading and lagging strand templates [57] but Pif1 is essential only for efficient replication through lagging strand G4s [60]. Consequently, the distinct behaviour of Pif1 at the leading and the lagging strands may be due to different conformations of the respective G-quadruplexes, affecting their folding and/or their processing due to the occupancy rate of RPA. Another possibility could be that the blocking G-quadruplex structures are better tolerated and bypassed by the lagging strand replication machinery due to its ability to prime DNA synthesis downstream of G-quadruplexes. However, this seems improbable because CEB1 instability in *rfa1-D228Y* cells is fully suppressed by Pif1 overexpression suggesting that the blocking structures are present and that the helicase activity of Pif1 is still required to remove G4-structures and rescue CEB1 stability.

How exactly Pif1 is recruited at the G-rich motifs and at G-quadruplexes that form at CEB1 during replication? One possibility is that Pif1 travels with the replication fork and facilitates replication by processing G-quadruplex structures at the leading-CEB1. Indeed, Pif1 interacts with PCNA and Cac1, the large CAF-1 subunit, which preferentially assembles nucleosome onto replicating DNA [60, 61]. Thus, Pif1 may be targeted to the replication fork by its ability to interact with PCNA or the histone chaperone Cac1 and preserves genome stability by acting at G-rich motifs at the leading-CEB1. Alternatively, Pif1 could be directly recruited to G-rich motifs and G-quadruplex structures. Mms1 is a G4-DNA-binding protein that helps replication fork progression at G4 and Pif1 binding to specific G4 structures [22, 37]. We show that Mms1 is not required to maintain CEB1 stability at both leading- and lagging-CEB1 revealing that G4 structures targeted by Mms1 are not deleterious for CEB1 stability. Our results are in good agreement with previous observations showing that Mms1 supports Pif1 function at G4 motifs only on the lagging strand [22, 37], whereas Pif1 is not required for CEB1 stabilisation [33]. Here we show that RPA recruits Pif1 to CEB1. We report that Pif1 associates with RPA and that this association is affected in the *rfa1-D228Y* mutant and by DNA digestion, suggesting that Pif1-RPA association relies on specific DNA structures. In addition we show that *rtt105*Δ mutation, which decreases the level of RPA associated with ssDNA (as the *rfa1-D228Y* mutation) [44, 45], strongly affects CEB1 stability at both leading and lagging strands. These results reveal the importance of Rtt105 in promoting the replication of G4-forming CEB1 minisatellite, during leading strand and lagging strand synthesis, and confirm that the level of RPA is crucial for CEB1 stability. RPA protects and stabilizes ssDNA susceptible to secondary structure formation. It is possible that the ability of RPA to directly bind to Pif1 could be important to recruit Pif1 at specific G-rich sequences/structures and/or to stimulate Pif1 activity at G-rich motifs and G-quadruplex structures.

Importantly, the results obtained in *S. cerevisiae* with the *rfa1-D228Y* mutant were confirmed in *S. pombe* with the *rpa1-D223Y* mutant. The effects of the *rpa1-D223Y* mutant were even more pronounced in *S. pombe*. This may be due to the fact that fission yeast lacks a functional ortholog of the Pif1 helicase. Indeed Pfh1 behaves more closely to the budding yeast Rrm3 helicase than to Pif1 [56]. This may account for the even more prominent role of RPA complex in G4 unwinding in fission yeast. This assumption is also supported by the fact that the *rpa1-D223Y* mutation has a stronger effect at telomeres in *S. pombe* than its counterpart in *S. cerevisiae* [38,62]. Interestingly, we observed expansions of the CEB25-L1T even in the WT strain, especially when CEB25-L1T was preferentially replicated by the lagging machinery. This result opens new avenues to use fission yeast as a model organism to study expansions of G-rich sequences that have been associated with neurological diseases.

RPA interacts with RNase H1 and colocalizes with it at R-loops raising the possibility that RPA could recruit and/or stimulate RNase H1 [41,53]. We observed that the interaction between RPA and RNase H1 is stimulated by the presence of DNA and is slightly affected in *rfa1-D228Y* cells. Furthermore, Pif1 regulates R-loop formation and potentially complements RNase H for R-loop resolution [51, 52]. We found that RNase H1 overexpression did not rescue CEB1 stability in *pif1*Δ, *rfa1-D228Y*, and *rtt105*Δ mutants. These results suggest that R-loops are likely not responsible of CEB1 instability in these mutants, indicating that CEB1 instability is not due to transcription. Collectively, our results add new insights about the role of RPA as a general sensor of secondary structures and regulator of genomic stability. RPA acts through direct interactions with proteins acting at the level of specific secondary structures, which are hotspots of genomic instability. This mode of RPA action is conserved between yeasts and humans.

## MATERIALS AND METHODS

### Strains and growth conditions

Strains used in this study are listed in Table 2. Yeast strains containing CEB1-1.8 I (orientation I), CEB1-1.8 II (orientation II), or CEB1Gmut-1.7 were mated with *pif1*Δ, *rfa1-D228Y, pif1*Δ *rfa1-D228Y, mms1*Δ, or *rtt105*Δ mutants. After sporulation and identification of the four resulting spores, the strains of interest were plated on rich medium at 30°C to obtain isolated colonies. Individual clones were grown in rich liquid culture at 30°C until stationary phase. For *PIF1* overexpression experiments, WT cells and *rfa1-D228Y* cells were transformed with the centromeric plasmids pVS45 (expressing the nuclear form of Pif1 under the control of the *GAL1* promoter) and pSH380 (a pRS315-derived vector control) provided by Virginia Zakian [43], then mated with leading-CEB1 (orientation I), or lagging-CEB1 (orientation II) cells. Individual clones were grown in SD-Leu (2% glucose), or SGal-Leu (2% galactose) media for repression or overexpression of *PIF1*, when mentioned.

**Table 2.** Strains used in this study.

		
***S. cerevisiae* strains**	**Genotype**	**Origin**
ORT6119-4	*MATα CEB1-1.8 I-ARS305*	Nicolas A.
ORT6135-36	*MATα CEB1-1.8 II-ARS305*	Nicolas A.
ORT6157-1	*MATα CEB1-1.7 Gmut-ARS305*	Nicolas A.
LM361	*diploïd CEB1-1.8 I rfa1-D228Y/RFA1 pif1::Kan/PIF1*	This study
LM411	*diploïd CEB1-1.8 II rfa1-D228Y/RFA1 pif1::Kan/PIF1*	This study
LM349	*diploïd CEB1-1.7 Gmut rfa1-D228Y/RFA1 pif1::Kan/PIF1*	This study
LM396	*diploïd CEB1-1.8 I rfa1-D228Y/RFA1 + pVS45* (GAL::*PIF1*)	This study
LM398	*diploïd CEB1-1.8 II rfa1-D228Y/RFA1 + pVS45* (GAL::*PIF1*)	This study
LM401	*diploïd CEB1-1.8 I rfa1-D228Y/RFA1 + pSH380* (empty vector)	This study
LM404	*diploïd CEB1-1.8 II rfa1-D228Y/RFA1 + pSH380* (empty vector)	This study
YVC600	*diploïd CEB1-1.8 I mms1::TRP1/MMS1*	This study
YVC601	*diploïd CEB1-1.8 II mms1::TRP1/MMS1*	This study
YVC602	*diploïd CEB1-1.8 I rtt105::Kan/RTT105*	This study
YVC603	*diploïd CEB1-1.8 II rtt105::Kan /RTT105*	This study
YVC604	*diploïd CEB1-1.8 I rtt105::Kan/RTT105* GAL*::RNH1::natMX/RNH1*	This study
YVC605	*diploïd CEB1-1.8 II rtt105::Kan/RTT105* GAL*::RNH1::natMX /RNH1*	This study
YVC606	*diploïd CEB1-1.8 I pif1::Kan/PIF1* GAL*::RNH1::natMX /RNH1*	This study
YVC607	*diploïd CEB1-1.8 I rfa1-D228Y/RFA1* GAL*::RNH1::natMX /RNH1*	This study
YVC608	*diploïd CEB1-1.8 II rfa1-D228Y/RFA1* GAL*::RNH1::natMX /RNH1*	This study
LM140	*MATα rfa1-D228Y Pif1-myc::KanMX6*	This study
W1042-7C	*MATα can1-100,x SUP4-****o****::HIS3::pWJ317-CAN1-URA3 rfa1-D228Y*	Rothstein R
LM301	*pif1::KanMX6 rfa1-D228Y*	This study
LM340	*pif1::KanMX6*	This study
YBL103	*his3Δ1; leu2Δ0; ura3Δ0; met15Δ0; pGal-3HA-RNH1::NAT*	Luke B.
LM407	*diploïd pGal-3HA-RNH1::natMX rfa1-D228Y/RFA1*	This study
***S. pombe strains***	**Genotype**	**Origin**
JA961	*h- leu1-32 ura4-D18 leu1+::pJK148-CEB25* orientation 1	This study
JA963	*h- leu1-32 ura4-D18 leu1+::pJK148-CEB25-G12T* orientation 1	This study
JA947	*h- leu1-32 ura4-D18 leu1+::pJK148-CEB25* orientation 2	This study
JA948	*h- leu1-32 ura4-D18 leu1+::pJK148-CEB25-G12T* orientation 2	This study
SC387	*h+ leu1-32 ura4-D18 ade6-M210 rad11-D223Y*	Ueno M.

All LM strains used are derivatives of W303-1B. All YVC strains used are derivatives of W303.

Spore colonies were generated from the diploid strains.

The 0.62 kb CEB25-L1T and CEB25-L1T-G12T were cloned into pJK148 integrative plasmid in both orientations at *Not*1 site, from plasmid pPA84 and pPA84-G12T (A. Nicolas's Laboratory), respectively. The corresponding plasmids were linearized by *Nde*1 and transformed into *S. pombe* cells. To check the insertion of CEB25 at the *leu1* locus, genomic DNA was digested by *Xba*I a nd southern blotted using a *leu1* probe. Correct and unique insertion of CEB25 at this locus generated two fragments of 3 and 14.4 kb. To check the correct size of CEB25-L1T and CEB25-L1T-G12T, a second digestion by PvuII on genomic DNA was performed on selected clones. Southern blot hybridized by a CEB25-L1T probe (*Not*1-fragment from pPA84 plasmid), revealed a fragment of 1.1 kb. Fission yeast strains containing CEB25-L1T and CEB25-L1T-G12T were then mated with the *rpa1-D223Y* mutant. After sporulation and identification of the resulting spores, the stability of the CEB25 was controlled a second time by Southern blot after digestion of genomic DNA by *Pvu*II. The strains of interest were plated on rich medium to obtain isolated colonies and individual clones were further grown in rich liquid culture at 32°C until stationary phase.

### Southern blot analyses

Genomic DNA was prepared from 1x10^8^ cells according to standard protocols and digested with *Apa*I/*Xho*I, *Apa*I/*Nco*I and *Apa*I/*Sac*II (New England Biolabs) for analysis of CEB1-1.8 I, CEB1-1.8 II and CEB1Gmut-1.7, respectively [31, 63]. The digested DNA was resolved in 1% agarose gel and blotted onto Hybond-XL membrane (GE Healthcare). After transfer, the membrane was cross-linked with UV and hybridized with CEB1-0.6 and CEB1 Gmut probes for CEB1-1.8 and CEB1Gmut -1.7, respectively. ^32^P labelling of DNA probes was performed by random priming using Klenow fragment exonuclease (New England Biolabs), in presence of [α-^32^P]-CTP and hybridizations were performed in Church buffer at 55°C. Radioactive signals were detected using a BIORAD molecular imager FX.

### Co-Immunoprecipitation experiment

Yeast cells were grown at 30°C in YPD to OD_600_=0.8. Extracts were lysed with glass-beads in TMG-50 (10mM TrisHCl pH8.0, 1mM MnCl_2_, 10% (v/v) glycerol, 50 mM NaCl, 0.1 mM DTT) containing a protease inhibitor cocktail (Calbiochem), MG132 (Sigma Aldrich), and 0.5% (v/v) Tween-20. Pif1-Myc and Rnh1-HA proteins were immunoprecipitated with 9E10 monoclonal anti-Myc antibody (Santa Cruz, CA) and 12CA5 anti-HA monoclonal antibody (Roche), respectively. The presence of Rfa1 and Rfa2 in the IP was checked with anti-Rfa1 and anti-Rfa2 antibodies (Agrisera, Sweden).

### RNA isolation and analysis

Total RNA was isolated from 2x10^8^ cells using the hot phenol method [64]. Total RNA was treated with RNase-free DNase I (Qiagen) and reverse transcribed using the SuperscriptIII reverse transcriptase (Life technologies) with random hexanucleotide primers (Sigma-aldrich). Quantitative PCR amplification of cDNA was carried out using the SYBR Premix Ex Taq II (Ozyme) with these cycling parameters: 1 cycle at 95°C for 30 sec, followed by 45 cycles at 95°C for 10 sec, 58°C for 15 sec, and 72°C for 20 sec, and a final extension step at 72°C for 5 min. *PIF1* cDNA was quantified by using the oligonucleotides Pif1-forward (5′-CTGAAAACTCATTTGACCAG-3′) and Pif1-reverse (5′-GCAATCTTTTCTCCAAATTGC-3′). *ACT1* cDNA was quantified by using the oligonucleotides Act1-forward (5′-CTATGTTACGTCGCCTTGGA-3′) and Act1-reverse (5′-TTTGGTCAATACCGGCAGAT-3′).

## Abbreviations:

Co-IP: – Co-immunoprecipitation,
RPA: – replication protein A,
ssDNA: – single-stranded DNA,
WT: – wild type.
